# Shorter Antibacterial Peptide Having High Selectivity for *E. coli* Membranes and Low Potential for Inducing Resistance

**DOI:** 10.3390/microorganisms8060867

**Published:** 2020-06-08

**Authors:** Adriana Barreto-Santamaría, Zuly Jenny Rivera, Javier Eduardo García, Hernando Curtidor, Manuel Elkin Patarroyo, Manuel Alfonso Patarroyo, Gabriela Arévalo-Pinzón

**Affiliations:** 1 Receptor-Ligand Department, Fundación Instituto de Inmunología de Colombia (FIDIC), Carrera 50#26-20, Bogotá 111321, Colombia; adrianasantamaria10@gmail.com (A.B.-S.); mepatarr@gmail.com (M.E.P.); 2 Animal Science Faculty, Universidad de Ciencias Aplicadas y Ambientales (U.D.C.A.), Calle 222#55-37, Bogotá 111166, Colombia; 3 Chemistry Department, Sciences Faculty, Universidad Nacional de Colombia, Carrera 45#26-85, Bogotá 111321, Colombia; zjriveram@unal.edu.co; 4 Pharmacy Department, Sciences Faculty, Universidad Nacional de Colombia, Carrera 45#26-85, Bogotá 111321, Colombia; jaegarciaca@unal.edu.co; 5 Vicerrectoría de Investigación, Universidad ECCI, Carrera 19#49-20, Bogotá 111311, Colombia; hercur@gmail.com; 6 School of Medicine, Universidad Nacional de Colombia, Carrera 45#26-85, Bogotá 111321, Colombia; 7 School of Medicine and Health Sciences, Universidad del Rosario, Carrera 24#63C-69, Bogotá 112111, Colombia; mapatarr.fidic@gmail.com; 8 Molecular Biology and Immunology Department, Fundación Instituto de Inmunología de Colombia (FIDIC), Carrera 50#26-20, Bogotá 111321, Colombia

**Keywords:** antimicrobial peptide, minimum inhibitory concentration (MIC), minimum bactericidal concentration (MBC), minimum haemolytic concentration (MHC), therapeutic index (TI)

## Abstract

Antimicrobial peptides (AMPs) have been recognised as a significant therapeutic option for mitigating resistant microbial infections. It has been found recently that *Plasmodium falciparum*-derived, 20 residue long, peptide 35409 had antibacterial and haemolytic activity, making it an AMP having reduced selectivity, and suggesting that it should be studied more extensively for obtaining new AMPs having activity solely targeting the bacterial membrane. Peptide 35409 was thus used as template for producing short synthetic peptides (<20 residues long) and evaluating their biological activity and relevant physicochemical characteristics for therapeutic use. Four of the sixteen short peptides evaluated here had activity against *E. coli* without any associated haemolytic effects. The 35409-1 derivative (17 residues long) had the best therapeutic characteristics as it had high selectivity for bacterial cells, stability in the presence of human sera, activity against *E. coli* multiresistant clinical isolates and was shorter than the original sequence. It had a powerful membranolytic effect and low potential for inducing resistance in bacteria. This peptide’s characteristics highlighted its potential as an alternative for combating infection caused by *E. coli* multiresistant bacteria and/or for designing new AMPs.

## 1. Introduction

Bacterial resistance represents a serious threat to worldwide public health; pathogens resistant to currently available antibiotics are becoming more common [1]. It is quite obvious that if suitable strategies are not introduced, then this problem will continue to grow, leading to an expected ten million deaths annually by 2050 [2]. Since it was discovered that variable length sequence (5 to 100 residues) antimicrobial peptides (AMPs) form part of almost all organisms’ innate immune response [3], they have been explored and exploited as alternatives for combating microbe-related infections. This has been due to their broad spectrum of antimicrobial activity and the combination of multiple mechanisms of action and rapid activities minimising the possibility of developing resistance [4,5]. AMPs can act against bacteria, fungi, viruses, parasites and cancer cells, acting on membrane and/or anionic targets in cell wall or inside them [5,6]. The electrostatic interaction between AMPs’ cationic amino acids (aa) and microbial membranes’ negatively charged components has been proposed as crucial for AMP activity. Although AMPs may have different mechanisms of action, it is thought that their ability to act against such diverse cellular organisms is related to membrane activity [7]. Lipid bilayer disruption models have been proposed to explain such membrane activity, i.e., toroidal-pore wormhole, carpet, barrel-stave models and detergent-type membrane lytic mechanism [6,8]. For example, LL-37 and pexiganan, or MSI-78, disrupt bacterial membrane by forming a toroidal pore [9,10].

Many structure–activity relationship (SAR) studies have compiled useful information about the physicochemical properties of the peptide sequences governing their activity and selectivity by microorganisms [11], such as their length, charge, structure, hydrophobicity and amphipathicity (i.e., an ability to assume an amphipathic α-helix conformation which is intimately related to AMP activity) [12,13].

Although many peptides isolated from natural sources have powerful antimicrobial activity, they frequently have certain limitations related to their toxicity against mammalian cells, high production costs and poor stability and/or bioavailability in host physiological conditions [14,15]; synthetic peptides have thus aroused interest. In addition to isolating natural AMPs [16], synthetic AMPs can be obtained by recombinant expression [17] or chemical synthesis [18]; the latter has become popular due to its advantages such as extremely pure products being able to be obtained rapidly and efficiently [19]. 

Many peptide candidates are currently undergoing preclinical and clinical studies [20]. However, some of them have been limited due to in vivo model results not reflecting those suggested by the promising results of in vitro studies, and even clinical trial results do not reflect preclinical studies’ results in animal models [21]. Iseganan (protegrin-1 analogue) is a case in point as it failed in phase III clinical trials as a mouthwash for reducing mucositis and stomatitis (oral mucositis) in chemotherapy patients [22]. XMP.629 is another example as it failed to demonstrate its efficacy for treating acne in phase III clinical trials along with Omiganan, which failed to prevent or reduce venous catheter-related bloodstream infections [21]. This also happened with pexiganan, the first commercially developed AMP. Pexiganan is a magainin-derived peptide which had shown promising results in several studies for treating diabetic foot infections [23,24]; however, it required very high doses in an animal model to be effective and failed to demonstrate therapeutic advantages over agents available for approval by the US Food and Drug Administration (FDA) [25]. 

Despite such disappointing results, AMPs still hold the spotlight as new generation antibiotics due to their versatility regarding being improved by chemical synthesis. Seven AMPs have been approved by the FDA, with their half-lives ranging from 5 h to 14 days. Vancomycin and its derivative dalbavancin (i.e., a lipoglycopeptide) inhibit bacterial wall synthesis, while its derivatives oritavancin and telavancin have a dual membranolytic and wall synthesis inhibition mechanisms. Gramicidin D is a pore-forming linear peptide whose half-life is unknown. Daptomycin is a membranolytic, cyclic lipopeptide and colistin or polymyxin E is another membranolytic, cyclic lipopeptide [26]. 

Apart from these AMPs, the FDA has approved many more peptides for other therapeutic applications such as treating diabetes and/or cancer [26]. The related findings highlight peptide engineering’s fundamental role in obtaining optimised sequences in laboratories, having a place in a therapeutic setting [15]. 

Such optimisation would include chemical synthesis, as it enables a broad range of modifications to be made to peptide design for improving therapeutic properties such as cyclisation or introducing non-natural amino acids (aa) or non-peptide portions [15,27]. Several researchers have adopted a chemical synthesis-based approach to obtaining shorter antimicrobial derivatives as this can increase selectivity and stability while reducing production costs [3]. Chemical synthesis is shown to be robust in this type of study as it enables a diversity of different length sequences to be obtained rapidly, thereby having different physicochemical characteristics [28].

A 20 aa-long *P. falciparum* Rifin protein-derived peptide *Pf*Rif (^321^RYRRKKKMKKALQYIKLLKE^340^), also known as peptide 35409, in which lysine has been replaced by alanine in position 331, has been shown to have antibacterial activity against *Escherichia coli* ML35 at 22 µM minimum inhibitory concentration (MIC). This peptide did not have cytotoxic activity against HeLa and HepG2 human cell lines, but did have activity against human red blood cells (hRBC) at 1.5 µM minimum haemolytic concentration (MHC). Its therapeutic index (TI) calculated for *E. coli* ML35 is 0.045, indicating low selectivity [29], thereby restricting its therapeutic use [30]. Considering the 35409 sequence’s antibacterial potential and the urgent need for developing new molecules having activity against microorganisms, this research was focused on obtaining short 35409-derived synthetic peptides having high selectivity for microorganisms. This led to selecting 16 synthetic peptides for various antibacterial and haemolytic assays. Four derivatives proving active against *E. coli* and inactive against hRBC were thus identified by bioinformatics analysis, structural data and functional assays. Among them, peptide 35409-1, which adopted an α-helical conformation in sodium dodecyl sulphate (SDS), showed the greatest antibacterial potential against ATCC strains and clinical isolates. 

This study led to the obtaining of a 17 residue long sequence having a selective membranolytic effect against *E. coli* cells, stable activity in the presence of human sera and low potential for inducing bacterial resistance. Such characteristics suggested that peptide 35409-1 could be a candidate for combating multiresistant bacterial infections caused by *E. coli* and/or designing new AMPs. 

## 2. Materials and Methods

### 2.1. 35409-Derived Peptide Synthesis

Short peptides derived from 35409 were obtained by solid-phase peptide synthesis (SPPS) using the Fmoc/tBu strategy [31,32]. This involved using a Rink amide resin (100 mg, 0.46 meq/g substitution). Deprotection involved removing the Fmoc group by double treatment with a 25% 4-methylpiperidine, 1% triton X-100 in N,N-dimethylformamide (DMF) (*v/v*) solution at room temperature (RT) with constant shaking (CS) for 10 min. Following four washes with DMF, 2-isopropanol (IPA) and dichloromethane (DCM), Fmoc-aa activation and coupling reactions were carried out using carbodiimide and the modified ester strategy. N,N’-Dicyclohexylcarbodiimide (DCC) and 1-hydroxy-6-chlorobenzotriazole (6-Cl-HOBt) (1:1:1; 5 excesses regarding resin meq) were dissolved in DMF for 15 min at RT with CS. Activated aa were mixed with the resin or resin-peptide and made to react for 12 h with CS at RT. The solution was removed by filtration and washed twice with DMF, IPA and DCM. The deprotection and coupling reactions were monitored by Kaiser test. 

The side chains were deprotected and the peptide cleaved by treating the resin-peptide with a cocktail of TFA/H_2_O/1,2-ethanedithiol(EDT)/triisopropylsilane (TIPS) (92.5/2.5/2.5/2.5% *v/v*) for 4–6 h at RT. EDT was added to this cocktail for controlling methionine residue oxidation [33]. The solution was filtered, and the peptide precipitated by adding cold ethyl ether. The solution was spun at 2500 rpm for 10 min and the precipitate washed five times with cold ethyl ether.

### 2.2. 35409-Derived Peptides’ Purification and Characterisation

The products were characterised by reverse-phase, high-performance liquid chromatography (RP-HPLC) and purified by solid-phase extraction (SPE) [32]. Peptide dissolved (1 mg/mL) in solvent A (0.05% TFA in water) was analysed on HPLC Agilent (series 1260) using a C18 column (Chromolith 4.6 × 50 mm) and 5% to 50% elution gradient solvent B (0.05% TFA in acetonitrile) in solvent A. Gradient time was 8 min at 2 mL/min flow rate, read at 210 nm. 

An elution gradient was designed for every peptide on a Supelclean LC-18 RP-SPE column based on its chromatographic profile. Peptides dissolved in solvent A were filtered through a 0.44 μm membrane and seeded in the column. Elution involved using a step gradient with increasing concentrations of solvent B; the fractions were collected and analysed by RP-HPLC, combining the fractions having the highest chromatographic purity [34].

A Bruker Daltonics Microflex LT mass spectrometer was used for determining the peptides’ mass/charge ratio by matrix-assisted laser desorption ionization-time of flight (MALDI-TOF) MS [30]. The peptide (1 mg/mL) was mixed with 1 mg/mL α-cyano-4-hydroxycinnamic acid in an 18:2.5 *v/v* ratio. One microlitre of this mixture was placed on the equipment’s sample plate and dried at RT. Readings were made in reflextron mode at 35–50% laser power using 200 shots.

### 2.3. Bioinformatics Analysis of the Peptide Sequences

The online APD3 antimicrobial peptide calculator and predictor (http://aps.unmc.edu/AP/) was used for predicting the sequences’ physicochemical characteristics [35]. HeliQuest online software (http://heliquest.ipmc.cnrs.fr/cgi-bin/ComputParamsV2.py) was used for measuring helix amphiphilicity/hydrophobic moment [36].

### 2.4. Circular Dichroism (CD)

Circular Dichroism (CD) was used for analysing the peptides at 5 µM final concentration, using a 1 cm optical path-length quartz cuvette in 1X PBS and 1X PBS with 10mM SDS as co-dissolvent [37,38,39]. A Jasco J-810 spectropolarimeter with nitrogen flow was used for obtaining the spectra, making an average of three sweeps at 260 to 190 nm at 20 nm/min exploration speed and one nm bandwidth. Spectra Manager Software was used for collecting the data [40].

### 2.5. Determining MIC by Broth Microdilution

The standard broth microdilution method was used for determining the peptides’ MIC [41]. Briefly, serial peptide dilutions (100 to 0.2 µM concentration) were incubated in 96-well plates at 100 μL/well final volume for 18 h at 37 °C in a damp chamber with ~5 × 10^5^ CFU/mL bacterial inoculum (calculated from the calibration curve equation previously obtained for each strain (Figure S1)). Optical density (OD) was measured at 620 nm after 18 h on a microplate reader [41,42]. Bacteria in Müller–Hinton broth (MHB) were used as negative control. The positive controls consisted of bacteria treated with template peptide 35409 [30] and bacteria treated with antibiotics (ciprofloxacin, gentamicin, ampicillin or vancomycin according to the bacterial strain). The MIC was determined as the lowest peptide concentration at which bacterial growth was not detected.

### 2.6. Bactericidal Activity

Bactericidal activity was evaluated by sowing an aliquot of Luria Bertani agar (LBA) in the wells where no growth had been detected in the MIC assay. Following 18 h incubation at 37 °C, the minimum bactericidal concentration (MBC) was determined as the lowest peptide concentration which could kill 99.9% of the bacteria, i.e., no growth in the agar [42].

### 2.7. Antibacterial Activity in the Presence of Human Sera 

The MIC was also determined by microdilution in broth for evaluating whether peptide stability and activity were affected by fresh human sera with no pre-incubation or pre-incubating the peptides with human sera for 6 h. The first assay involved peptide dilution with fresh human sera (100%) followed by incubation with a bacterial inoculum diluted in MHB (~5 × 10^5^ CFU/mL) for starting the MIC assay. The second assay involved peptide dilution in fresh human sera (100%) pre-incubated for 6 h at 37 °C in a humid chamber before putting them in contact with the bacterial inoculum diluted in MHB (~5 × 10^5^ CFU/mL). The assay was incubated and read as mentioned in the microdilution section.

### 2.8. Antibiotic Synergy

The checkerboard method was used for evaluating peptide antibacterial activity in combination with antibiotics [43]. The peptides at different concentrations (1:2 dilutions from 2 × MIC to 0.03 × MIC) in absence or in combination with different antibiotics and concentrations (gentamicin or ciprofloxacin) were added to a 96-well plate. The bacterial suspension was then added at ~5 × 10^5^ CFU/mL final concentration in MHB and incubated at 37 °C for 18 h. Absorbance was measured at 620 nm and individual peptide and antibiotic MIC and combined MIC were obtained. Method 3, reported by Bonapace (2002), was used for interpreting them [44]; fractional inhibitory concentration (FIC) was calculated by dividing the MIC in combination over individual MIC. The fractional inhibitory concentration index (FICI) was then calculated as the sum of peptide FIC + antibiotic FIC. A ≤ 0.5 FICI indicated synergy, 0.5 to ≤4 FICI indifference, and >4 FICI antagonism [45].

### 2.9. Haemolytic Activity

A haemolytic activity assay was used for determining whether peptides acted on hRBC membrane by calculating the minimum haemolytic concentration (MHC). Briefly, 100 µL serial dilutions of peptides were incubated in a 96-well plate with an equal volume of a fresh hRBC suspension at 5% *v/v* in 1X PBS (200–0.39 µM final peptide concentrations). The samples were spun at 1000× *g* for 5 min after having been incubated at 37 °C for 1 h and a Multiskan spectrometer (Thermo Fisher) was used for measuring 100 µL supernatant absorbance at 560 nm. Treated hRBC absorbance 0.1% Triton X-100 or 100% haemolysis was used as positive control [46]. 

### 2.10. Scanning Electron Microscopy (SEM)

SEM was used for evaluating the effect on *E. coli* ATCC 25922 cells caused by the most effective peptide derivative [47]. Briefly, 5 × 10^7^ CFU/mL was incubated with the peptides in 1X PBS at 37 °C for 1 h. The sample was washed twice with 1X PBS and suspended in 2.5% glutaraldehyde for fixing the bacteria. The samples were dehydrated gradually using ethanol at 70% to 100% concentration. The samples were mounted on metal pins (no carbon membrane), air-dried and gold plated using a Quorum Q150R ES metaliser. Untreated bacteria were used as negative control. The samples were analysed by FEI Quanta 200 SEM at 25 kV.

### 2.11. Permeabilising E. coli ML35 Internal Membrane 

The ML35 strain (constitutively producing β-galactosidase) was sown in triplicate in 96-well plates (~5 × 10^7^ CFU/mL) in the presence of peptides at 1× MIC in 1X PBS supplemented with 1.5 mM ortho-nitrophenyl-β-D-galactoside (ONPG) substrate at 100 µL final volume for ascertaining the most effective peptide derivative on *E. coli* internal permeability. The plates were incubated at 37 °C and o-nitrophenol production (yellow) was monitored every 30 min by spectrophotometry at 405 nm for 4.5 h [48]. Bacteria treated with cecropin P1 and bacteria treated with original peptide 35409 were used as permeabilisation positive control. Untreated bacteria and ciprofloxacin-treated bacteria were used as permeabilisation negative control. 

### 2.12. Evaluating the Potential for Inducing Bacterial Resistance in E. coli ATCC 25922

The MIC against *E. coli* ATCC 25922 was initially determined by microdilution in broth. After 18 h incubation, the bacteria treated with 0.5 peptide MIC (first well having bacterial growth detected by absorbance) were sub-cultured in MHB medium for ~4 h at 37 °C with constant shaking (~250 rpm). The MIC against this subculture was then determined by micro-dilution in broth. This was repeated consecutively for 18 days to determine the development of resistance by *E. coli* ATCC 25922 caused by continuous peptide treatment. Ciprofloxacin- and tetracycline-treated bacteria were used as controls [49]. Two independent assays were carried out in duplicate. MIC changes were plotted for each subculture (day of assay).

### 2.13. Ethics Statement

Blood samples for obtaining human RBC and sera were obtained from healthy subjects who had provided their written informed consent for inclusion in the study, which was carried out in line with the Declaration of Helsinki guidelines. The protocol was approved by the Universidad del Rosario’s Research Ethics Committee: approval code DVO005 782CV-1095.

## 3. Results

### 3.1. 35409-Derived Sequences

Sixteen 7- to 17-aa-long peptides derived from the 35409 sequence were designed and synthesised: seven truncated sequences from the N-terminal extreme (peptides 35409-1 a 7), five truncated sequences from the C-terminal extreme (peptides 35409-8 to 12) and four truncated sequences simultaneously from the N-terminal and C-terminal extremes (peptides 35409-13 to 16). All synthesized peptides contain an amide group at C-terminal end (–CONH_2_) (Table 1).

SPPS and RP-SPE purification led to obtaining peptides having a chromatographic purity shown in Table S1. Most peptides evaluated here had 86% to 99% purity. Mass spectra gave an m/z signal very close to that expected for the species ${\left[M+H\right]}^{+}$ in all cases, thereby corroborating each molecule’s identity. Figure S2 gives the results for peptide 35409-1 as an example.

### 3.2. Antibacterial and Haemolytic Activity

Antibacterial and bactericidal activity was initially evaluated by determining the peptides’ MIC and MBC regarding two Gram-positive strains and two Gram-negative strains. No peptide was active against Gram-positive *S. aureus* ATCC 25923 and *E. faecalis* ATCC 29212 strains at the concentrations evaluated here (0.2–100 µM) (Table 1). By contrast, some 35409-derived short peptides had activity against Gram-negative bacteria (Table 1). Peptides 35409-1 (17 residues) and -2 (16 residues) were capable of inhibiting bacterial growth against *E. coli* 25922 at the same concentration as that for original peptide (25 µM). Interestingly, short peptides’ (greater than 200 µM MHC) haemolytic activity became significantly reduced regarding original peptide 35409 MHC (1.56 µM).

### 3.3. The Peptide Sequences’ Physicochemical Properties 

The APD3 database was used for bioinformatics analysis of the sequences for studying the relationship between some of the peptides’ physicochemical characteristics and their biological activity. This showed that changes in peptide derivative length and charge varied from +2 to +10 and hydrophobic aa percentage from 0% to 50% (Table 1).

CD was used for studying secondary structure elements in 1X PBS (simulating physiological conditions) and in PBS with 10 mM SDS (mimicking bacterial surfaces’ negatively charged environment), as spectral differences have been observed in these media which could have been associated with AMP activity [50,51,52].

**Figure 1:**
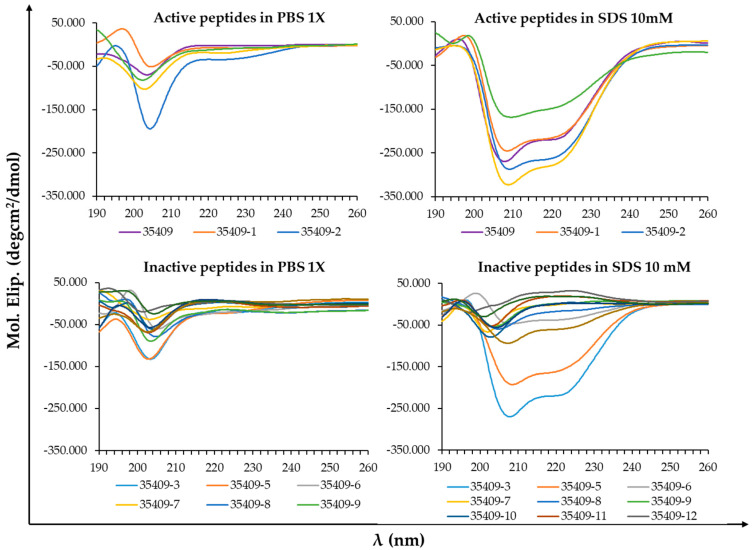
Circular Dichroism (CD) spectra for original peptide 35409 and its short derivatives. Possible secondary structure elements were studied in 1X PBS and 1X PBS with 10 mM SDS regarding peptides which were active or inactive against *E. coli*.

All the peptides had a disordered pattern in 1X PBS. The five peptides which were active against *E. coli* (original peptide and 35409-1, -2, -4 and -13 derivatives) had a spectral pattern characteristic of their α-helix structure in SDS in the 200 to 260 nm region, particularly regarding ~208 nm and ~222 nm negative signals [53]. Most inactive peptides had a structurally distorted trend in SDS, shown by a negative band between 195 and 210 nm [53]. Two of the 12 inactive peptides seemed to adopt a marked helical trend in SDS (peptides 35409-3 and -5) (Figure 1).

### 3.4. Antibiotic Synergy

MIC, MBC and MHC assay results led to using the checkerboard method for determining just 35409-1, -2, -4 and -13 synergic effect with two antibiotics widely used in clinical therapy: ciprofloxacin and gentamicin [54,55]. The results were interpreted by taking the lowest checkerboard FIC index (previously reported method 3) [44]. The FIC index gave indifference for all combinations in all cases (Table 2).

### 3.5. Spectrum of Activity against E. coli

The antibacterial activity of peptides 35409-1, -2, -4 and -13 was evaluated against other ATCC strains and *E. coli* clinical isolates. The four peptides had activity against three out of the four ATCC strains, having 3–25 µM MIC values, including activity regarding the ampicillin-resistant ATCC 35218 strain. No peptide was active against *E. coli* ATCC 11775 at the concentrations evaluated here (Table 3).

Approximately 50 bacterial samples were then collected from *E. coli*-infected patients for evaluating activity of the peptides against some clinical isolates. These samples were identified and biochemically and metabolically characterised using the VITEK-2 equipment. Isolates were profiled regarding their resistance or susceptibility to a panel of conventionally used antibiotics. Three *E. coli* isolates having different resistance profiles were then selected following such characterisation (Appendix A):

1. **Isolate 4 (from urine):** extended spectrum beta-lactamase (ESBL) (-), resistant to ampicillin, quinolones and co-trimoxazole.
2. **Isolate 40 (from blood):** ESBL (-), resistant to ampicillin, 1st-generation cephalosporins and co-trimoxazole.
3. **Isolate 44 (from blood):** ESBL (+), resistant to aminoglycosides, quinolones, β-lactams (including 1st-, 3rd- and 4th-generation cephalosporins).

It was found that peptides 35409-1, -2 and -13 were active against the two clinical isolates when evaluating their MIC, isolates having a higher resistance index regarding antibiotics (isolates N° 40 and 44), with MICs ranging from 12.5 to 50 µM (Table 3).

### 3.6. Activity in the Presence of Human Sera

Short peptide activity against *E. coli* ATCC 25922 was evaluated in the presence of human sera, and peptide stability was evaluated by pre-incubating the peptides with fresh human sera for 6 h before putting them in contact with the bacteria. No change in MIC in the presence of fresh human sera was found regarding MIC in MHB (Table 1 and Table 4). Original peptide activity and that for derivatives 35409-2, -4 and -13 became lost at the concentrations evaluated here when the peptides were pre-incubated for 6 h in human sera, while peptide 35409-1 (17 residue-long) maintained its activity (50 µM MIC) (Table 4). 

### 3.7. Mechanism of Action

**Figure 2:**
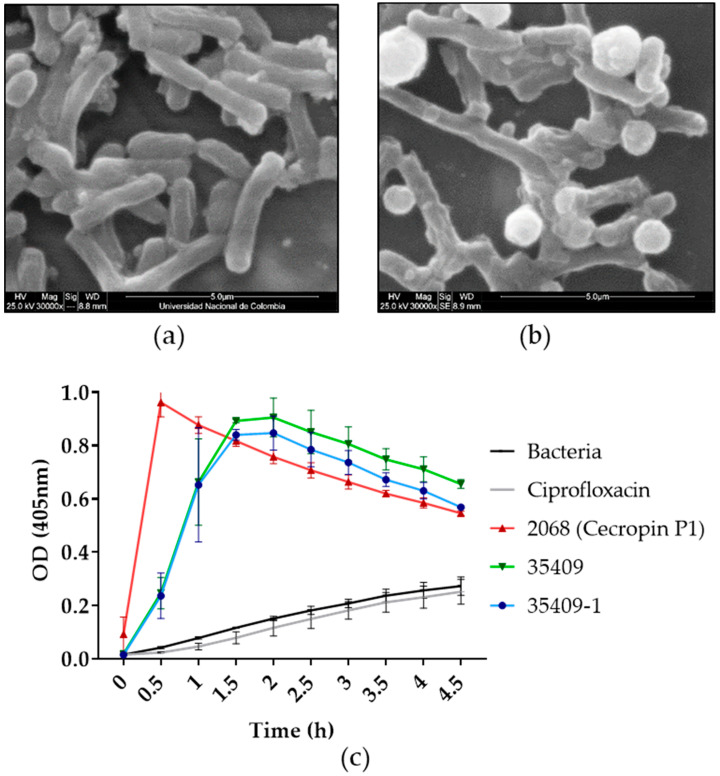
Peptide 35409-1 effect on *E. coli* membrane. Panels (**a**,**b**) show *E. coli* ATCC 25922, (**a**) without treatment and (**b**) treated with peptide 35409-1, by SEM. Panel (**c**) shows the permeabilisation of *E. coli* ML35 Gram-negative membrane evaluated with ONPG. The cecropin P1 peptide was used as positive permeabilisation control as it is recognised for its powerful membrane action. Untreated bacteria and ciprofloxacin-treated bacteria (an antibiotic having intracellular action) were used as negative controls.

SEM was used for evaluating the effect of derivative 35409-1 on *E. coli* morphology. Micrographs showed damage on the surface and leaking cytoplasmatic content in treated bacteria compared to untreated bacteria (Figure 2a,b). Such results coincided with those from permeabilisation assays where peptide 35409-1 did act on *E. coli* internal membrane, having similar permeabilisation kinetics to those for the previously published original 20 residue-long 35409 sequence [30]. Peptide 2068, representing the cecropin P1 peptide (positive permeabilisation control), was capable of inducing a high rate of permeabilisation for the first 30 min of incubation, thereby coinciding with the permeabilisation kinetics and bactericidal effect previously reported for this peptide [56]. Ciprofloxacin, an antibiotic acting against topoisomerase IV and DNA gyrase [57], did not have (as expected) any significant permeabilisation effect during the time the assay lasted.

### 3.8. Developing Resistance in E. coli ATCC 25922 Bacteria

35409-1’s potential for developing resistance was evaluated in *E. coli* ATCC 25922 and compared to two conventionally used antibiotics. Peptide 35409-1 had a lower MIC rate of change than that for conventional antibiotics, being capable of maintaining the same MIC for periods of up to 7–8 days in a row.

**Figure 3:**
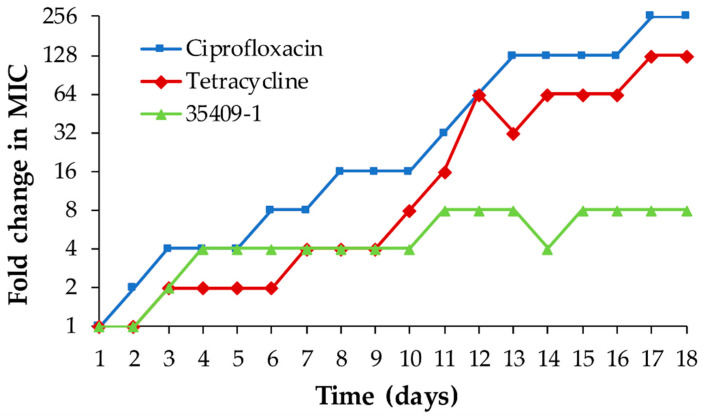
Developing resistance in *E. coli* ATCC 25922 by 35409-1-treatment. The figure shows MIC variation for bacteria repeatedly treated with 0.5× MIC for peptide 35409-1 compared to MIC variation for bacteria treated with 0.5× MIC for ciprofloxacin and tetracycline.

Consequently, although the antibiotics used had an up to 256-fold change in MIC after 18 days of study, 35409-1 only achieved an eight-fold increase in its MIC during the same time frame (Figure 3).

## 4. Discussion

AMPs represent a promising alternative for combating bacterial infections and pathogens developing resistance [58,59]. Identifying short sequences having selective antibacterial activity is thus relevant for overcoming certain limitations regarding the use of AMPs, mainly related to their synthesis, production costs and the possibility of scaling up [3]. This study has described the design and synthesis of short peptides derived from antibacterial peptide 35409 [30] and evaluated their activity against Gram-negative (*E. coli* and *P. aeruginosa*) and Gram-positive strains (*S. aureus* and *E. faecalis*). Their haemolytic activity was assessed, and the peptides characterised by bioinformatics and CD analysis. Four of the sixteen 35409-derived peptides had antibacterial activity solely targeting Gram-negative *E. coli* bacteria. Results for these derivatives agreed with those for the original 35409 sequence’s antibacterial activity, acting against Gram-negative *E. coli* ML35. The original peptide 35409 displayed greater activity against liposomes having high phosphatidylethanolamine content [30], such phospholipid occurring with greater abundance in Gram-negative bacteria than Gram-positive ones [60,61]; however, this particular characteristic was not assessed here for the modified derivatives.

The derivatives had no haemolytic effect at the concentrations evaluated here, suggesting a significant increase in their selectivity for bacterial cells compared to that for the original sequence (having haemolytic activity from 1.56 µM) (Table 1). This has been reported for other sequences where short AMP derivatives have been shown to have lower lytic activity against RBCs than that of their parent peptide, on occasions having increased or reduced antibacterial activity [62,63]. 

Peptides having 13 or less residues were seen to lose their antibacterial activity, indicating that a ≥35% reduction in length resulted in inactive peptides (Table 1). The sequences’ C-terminal regions were critical for peptide action, as removing three or more residues from this extreme led to a loss of antibacterial activity. It can be appreciated that increasing charge by removing a negatively charged residue can confer antibacterial activity on an inactive sequence by comparing inactive peptide 35409-3 (KKMKKALQYIKLLK**E**) sequence to that for 35409-13 (KKMKKALQYIKLLK), which was active against *E. coli* ATCC 25922. Antibacterial activity results suggested that 35409 N-terminal region RYR residues were not relevant for activity against *E. coli* strains, since derivative 35409-1 (which did not contain these three residues) had antibacterial and potential bactericidal activity against ATCC strains, similar to that for the original sequence and even more powerful against *E. coli* ML35 (Table 1 and Table 3).

Previous reports have suggested that a reduction in AMPs’ charge could reduce antibacterial activity [64]. However, peptides 35409-1 (+8) and 35409-2 (+7) maintained their antimicrobial activity despite a reduction in their charge, probably due to still being in the highest part of the range described for most AMPs (+2 to +9), thereby enabling them to become electrostatically attracted by negatively charged microbial surfaces [64,65]. 

The CD of the peptides dissolved in 1X PBS with or without SDS was used for comparing the structural trends of peptides active against *E. coli* ATCC 25922 to those for inactive peptides. Previous studies have made structural comparisons of AMPs in contact with SDS micelles versus AMPs in contact with phosphatidylethanolamine (POPE)/phosphatidylglycerol (POPG) liposomes by CD, suggesting similar structures in both preparations. Even Nuclear Magnetic Resonance (NMR) has been used with SDS for obtaining AMP structure and as the basis for simulating AMP-membrane interactions [66,67], showing the usefulness of SDS as an initial approach in conformational studies. 

CD spectra were obtained for the far ultraviolet region (180–250 nm) as signals in this region are very sensitive to conformational changes [68]. However, it has been argued that high chloride content in 1X PBS buffer creates noise in this region due to its strong absorption, meaning that the signal/noise ratio in some experiments is only acceptable up to 195–200 nm [69]. The peptides’ structural trends were thus only taken into account regarding the 200–260 nm spectral region due to such interference. All the sequences had a disordered conformation in 1X PBS, while the original peptide and the four active derivatives (35409-1, -2, -4 and -13) had negative signals at ~208 and ~222 nm in SDS micelles (Figure 1), such signals being characteristic of an α-helix pattern [53]. AMPs’ usual pattern consists of a disordered tendency in solution and the adoption of structural tendencies defined in a membrane or solvent environment simulating it, such as SDS [51,52,70]. A helix structure is the commonest one for AMPs, suggested here for all peptides acting against *E. coli*; for example, an α-helix structure is involved in 18.8% of the 41.5% of peptides having a known structure in the APD3 database (http://aps.unmc.edu/AP/statistic/statistic_structure.php). Peptide LL-37 and its short derivative KR-12-a5 provide a suitable example of such pattern as they have a disordered conformation in saline buffer and adopt an α-helix tendency in solvents containing SDS, TFE or LPS [51]. Two of the inactive sequences had a marked α-helix tendency, suggesting that although structural conformation is a relevant property, it is not the decisive factor for activity, and that activity and selectivity result from a set of multiple physicochemical properties as proposed by many studies [39,50,71,72,73].

Following initial evaluation, the study focused on short sequences having antibacterial activity, i.e., peptides 35409-1, -2, -4 and -13. Properties possibly being relevant for therapeutic use, such as antibiotic synergism, a spectrum of activity against other *E. coli* strains, activity against clinical isolates and stability in the presence of human sera, were thus evaluated. No peptide was synergistic for ciprofloxacin or gentamicin, showing that the combined administration of these antibiotics offered no therapeutic advantage; only an additive effect could be appreciated, i.e., the sum of individual activities [43,45].

**Figure 4:**
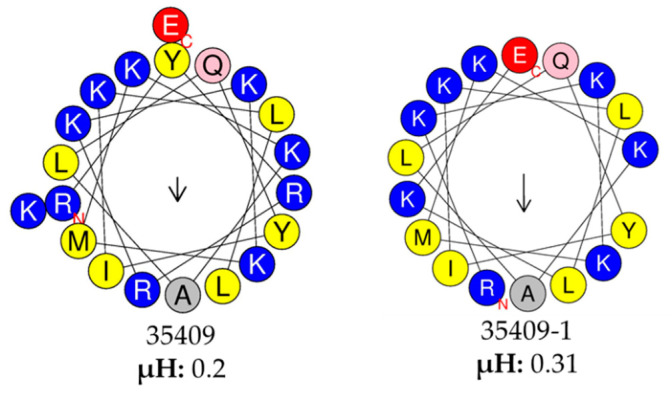
Helix representation and hydrophobic moment. The original peptide and peptide 35409-1 wheel projections are shown. The hydrophobic moment (µH) is shown in the lower part as a measure of amphipathicity. Yellow indicates non-polar amino acids and blue indicates polar amino acids. Arrow indicates helix non-polar face proportion and direction.

Peptides 35409-1, -2, -4, and -13 were active against other *E. coli* ATCC strains; however, none of them acted against *E. coli* ATCC 11775 (Serovar O1:K1:H7) (https://www.atcc.org › 11775). This could have been due to a capsular antigen, as the capsule has been proposed as a bait for AMPs, impeding their contact with bacterial membrane [74]. Peptides 35409-1, -2 and -13 acted against an ampicillin-resistant ATCC strain and two clinical multiresistant isolates (isolates 40 and 44) (Table 3 and Appendix A), thereby highlighting their clinical potential for combating bacteria having classical resistance mechanisms which have managed to overcome currently available antibiotic barriers [75,76]. Peptide 35409-1 has an interesting MIC regarding isolate 44 (12.5 μM) as it has been proposed that an antimicrobial must have a ≤ 16 μg/mL or ≤16 μM MIC as a requirement for being evaluated in clinical assays [77]. Peptide 35409-1 was the derivative having greater antibacterial activity and had no haemolytic activity, unlike the original sequence. Peptide 35409-1 had less charge and was shorter than its parent peptide whilst hydrophobicity and amphipathicity were greater (Table 1 and Figure 4). Changes of this type are associated with reduced selectivity as generic interaction with lipid membranes is favoured [15]; however, in our case such changes led to a sequence having less haemolytic potential and similar antibacterial potential.

The four derivatives’ (35409-1, -2, -4 and -13) activity against *E. coli* ATCC 25922 was maintained in the presence of fresh human sera. However, when the sequences were pre-incubated with human sera for 6 h, only peptide 35409-1 conserved its activity, suggesting the molecule’s greater stability in physiological conditions. This highlighted its potential use in clinical therapy as many AMPs lose their antimicrobial potential or become rapidly degraded in a targeted host and this has become one of the main limitations regarding their therapeutic use [51,78,79,80]. However, although human sera approaches bloodstream conditions, the residual proteases are not always representative of what occurs in the presence of RBC, where AMPs can become degraded by cytosolic proteases being released from RBC [81]. It should thus be interesting to continue evaluating 35409-1 stability in the presence of total blood and in in vivo conditions without underestimating this first in vitro approach’s scope.

The original sequence (35409) and peptide 35409-1’s TI were calculated by dividing the MHC by average MIC against ATCC *E. coli* strains. A > 17.5 TI for peptide 35409-1 indicated a significant increase in its selectivity regarding that for the original sequence as this was 220 times higher than that calculated for the original (0.08). 35409-1’s marked selectivity for *E. coli* bacteria and activity against multiresistant strains highlighted this peptide’s potential for being used as an antimicrobial. Moreover, this result led to an increase in AMPs arsenal against Gram-negative bacteria, considering that most AMPs described up to date target Gram-positive bacteria. Most AMPs are inactive against Gram-negative bacteria which could be related to lower negative charge concentration and these bacteria’s greater similarity with human cell membranes [14,15,20]. The forgoing arouses particular interest in peptide 35409-1 for use as an AMP, even more so as *E. coli* has been classified by the WHO as one of the species having critical priority regarding new antimicrobial research and development due to the increasing appearance of strains which are resistant to currently available antibiotics [82].

Peptide 35409-1’s possible effect on *E. coli* bacteria was explored by SEM. The micrographs showed that this peptide produced morphological changes on *E. coli* surface. Such changes involved loss of membrane continuity and intracellular material release, suggesting a membranolytic effect, the main mechanism of action described for AMPs against microorganisms [83]. Such mechanism of action was confirmed by using *E. coli* ML35 (bacteria having no lactose permease but constitutively forming β-galactosidase) and the ONPG substrate. Peptide 35409-1’s permeabilisation kinetics were similar to those of the original sequence with maximum permeabilisation being reached at 1.5 h, indicating this peptide’s capability for perforating *E. coli*’s internal membrane and coinciding with the reported bactericidal effect (Table 1). Such membrane permeabilisation action differed from antibiotics’ classical mechanism of action (usually affecting specific intracellular targets or those in the wall), which could explain why peptide 35409-1 acted against bacterial isolates, which have developed resistance against many conventional antibiotics [84]. 

Along with the peptide’s helical conformation (when using SDS), SEM and ONPG assays suggested membranolytic action. In general, AMP–membrane interactions are complex, requiring biophysical analysis. Several approaches have been used for extracting such information; isothermal titration calorimetry (ITC), bioinformatics simulations and NMR have revealed very precise information about AMP-membrane interactions [47,66,67,85,86,87,88]. For example, NMR has revealed interactions altering membrane curvature leading to its disruption for peptides such as those derived from magainin and LL-37 [9,10,88,89,90]. This indicated that additional studies with peptide 35409-1 are required to better understand its disposition on the membrane and the specific interactions taking place there, even though this study involved an initial approach to its mechanism of action on *E. coli* membrane.

AMPs’ lower potential for inducing/creating antimicrobial resistance has been one of the most mentioned reasons for defending their development as alternative therapeutic agents to antibiotics [59]. This study has evaluated peptide 35409-1’s potential for creating resistance regarding *E. coli* ATCC 25922 and compared it to that of two conventionally used antibiotics: ciprofloxacin, inhibiting topoisomerases II and IV (necessary for bacterial DNA replication, transcription, reparation and recombination) [57], and tetracycline, binding to ribosome inhibiting protein synthesis [91]. 

35409-1 MIC increased on the fourth day of the assay, reaching an MIC eight-fold higher than the initial reading on day 11 and maintained this until day 18. Antibiotics had a faster increase in MIC than 35409-1. Tetracycline reached an MIC 128-fold greater than its initial MIC by the end of the trial (day 18) and ciprofloxacin reached a MIC 256-fold greater than its initial MIC. This suggested that peptide 35409-1’s membranolytic effect might have been associated with the microorganisms having less opportunity to create resistance against it, mainly due to its rapid and non-specific electrostatic interaction with the membrane’s anionic components. This represents a high metabolic cost for microorganisms that would have to undergo a robust change in membrane composition to become resistant, instead of producing point mutations in a specific target for action [7,92]. This could be related with previous results where cationic AMPs acting on membrane did not accelerate *E. coli* mutation rate as occurs with antibiotics such as ciprofloxacin [93]. 

The results from this study have highlighted peptide 35409-1’s characteristics, suggesting that it could be a promising candidate for treating multiresistant infection caused by *E. coli* strains. However, it is clear that there is still a long way to go and serious limitations to be overcome regarding the clinical application of a new AMP [15,94]. Further studies must thus be carried out with peptide 35409-1 for better understanding its scope and limitations; this should also improve its profile for therapeutic use from an early development stage, i.e., in the laboratory, to avoid hasty transfer to preclinical and clinical stages that may lead to another therapeutic failure [95]. Animal models must be included to demonstrate 35409-1’s ability in in vivo conditions and rule out toxic effects. Even more so when it has been seen that a significant amount of AMPs called “promising” in the laboratory have then revealed their ineffectiveness when acting in in vivo models or requiring a higher than expected dose, sometimes very close to a toxic dose [23,24,25,94].

It is also interesting to note that 35409-1, having an average 14.3 µM MIC against *E. coli* (ATCC strains and clinical isolates), did not induce haemolysis at 14-fold higher concentrations. This improved peptide has thus overcome early on one of the original peptide’s most important limitations and that of AMPs in general, i.e., low selectivity [8,95]. It would be interesting to evaluate 35409 and 35409-1 interaction with bacterial membranes and mammalian membranes, as has been done for magainin 2 and its derivatives [96,97,98] to explore in depth whether such interaction is related to peptide 35409-1’s selectivity.

## 5. Conclusions

Many studies have emphasised the need for optimising peptide sequences from early stages onward to reduce the chance of AMPs’ therapeutic failure [15]. Stability, length and selectivity are characteristics to be optimised by peptide engineering [8,25,77]. This study has presented 17 residue long peptide 35409-1 (RKKKMKKALQYIKLLKE), an improved sequence obtained from peptide 35409, which was obtained by chemical synthesis, having 86–95% chromatographic purity, truncating the N-terminal region. This peptide, being shorter and having less charge but greater hydrophobicity and amphipathicity than the original sequence, had antibacterial activity against *E. coli* and *P. aeruginosa*, including an ampicillin-resistant *E. coli* ATCC strain. This peptide sequence acted against *E. coli* multiresistant isolates and maintained its activity after having been pre-incubated with human sera for 6 h. The peptide’s activity seemed to be highly selective for Gram-negative *E. coli* bacteria as it did not act against Gram-positive ones or hRBC. Its mechanism of action was based on *E. coli* cytoplasmatic content leakage by bacterial membrane permeabilisation. AMP–membrane interactions have been shown to have profound implications regarding AMPs therapeutic usefulness [97,98]; five of the seven FDA-approved AMPs are active on membrane [26], so this mechanism of action must be explored in depth for 35409-1.

Peptide 35409-1 had a considerably lower potential for creating/inducing resistance than conventionally used antibiotics. These results indicated that peptide 35409-1 could be a potential candidate for use in clinical therapy or developing new, highly selective AMPs targeting Gram-negative *E. coli*. Its stability in the presence of sera, activity against multiresistant *E. coli* bacteria, and its poor potential for inducing resistance suggest its significant therapeutic advantages for confronting the current problem regarding antibacterial *E. coli* resistance. 

Even though multiple studies are still lacking to ascertain whether improved 35409-1 could be used in the therapeutical industry [15,25], these first laboratory results have shown it to be a molecule having a good profile in vitro, removing some of AMPs’ most common limitations related to their length, selectivity and stability in serum.

## Figures and Tables

**Table 1:** 35409-derived peptides’ physicochemical characteristics and antibacterial activity. The peptides’ minimum inhibitory concentration (MIC), minimum bactericidal concentration (MBC) and minimum haemolytic concentration (MHC) are expressed in µM.

Peptide	Sequence	Physicochemical Characteristics			MIC/MBC ^1^		MHC ^2^
		L	Net Charge	% H aa	*E. coli* 25922	*P. aeruginosa* 27853	
35409	RYRRKKKMKKALQYIKLLKE	20	+10	30	25/50	>100	1.56
35409-1	RKKKMKKALQYIKLLKE	17	+8	35	25/25	100/100	>200
35409-2	KKKMKKALQYIKLLKE	16	+7	37	25/25	>100	>200
35409-3	KKMKKALQYIKLLKE	15	+6	40	>100	>100	>200
35409-4	KMKKALQYIKLLKE	14	+5	42	100/100	>100	>200
35409-5	MKKALQYIKLLKE	13	+4	46	>100	>100	>200
35409-6	ALQYIKLLKE	10	+2	50	>100	>100	>200
35409-7	YIKLLKE	7	+2	42	>100	>100	>200
35409-8	RYRRKKKMKKALQYIKL	17	+10	29	>100	>100	>200
35409-9	RYRRKKKMKKALQY	14	+9	21	>100	>100	>200
35409-10	RYRRKKKMKKA	11	+9	18	>100	>100	>200
35409-11	RYRRKKKMKK	10	+9	10	>100	>100	>200
35409-12	RYRRKKK	7	+7	0	>100	>100	>200
35409-13	KKMKKALQYIKLLK	14	+7	42	50/50	>100	>200
35409-14	KKMKKALQYIKL	12	+6	41	>100	>100	100
35409-15	RKKKMKKALQY	11	+7	27	>100	>100	200
35409-16	KMKKALQY	8	+4	37	>100	>100	>200
**C+**	**ciprofloxacin MIC (µg/mL)**	**-**	**-**	**-**	**0.008**	**0.256**	**-**

L: length, %H aa: percentage of hydrophobic amino acids; ^1^ minimum inhibitory concentration and minimum bactericidal concentration; ^2^ minimum haemolytic concentration. All synthesized peptides contain an amide group at C-terminal end (–CONH_2_), this increases the charge of the peptides by +1.

**Table 2:** Synergy with conventional antibiotics.

Antibiotic (A)	Peptide (P)	x MIC Individual/Combination		FIC P + A	FIC Index	Interpretation
		Peptide	Antibiotic			
**Ciprofloxacin**	**35409-1**	1/1	1/2	1 + 2	3	Indifference
	**35409-2**	1/1	1/2	1 + 2	3	Indifference
	**35409-4**	1/1	1/2	1 + 2	3	Indifference
	**35409-13**	1/1	1/2	1 + 2	3	Indifference
**Gentamicin**	**35409-1**	1/0.25	1/0.5	0.25 + 0.5	0.75	Indifference
	**35409-2**	1/0.25	1/0.5	0.25 + 0.5	0.75	Indifference
	**35409-4**	1/0.25	1/0.5	0.25 + 0.5	0.75	Indifference
	**35409-13**	1/0.25	1/0.5	0.25 + 0.5	0.75	Indifference

**FIC:** fractional inhibitory concentration. **Individual ciprofloxacin MIC:** 0.008 µg/mL. **Individual gentamicin MIC:** 0.5 µg/mL. Values are given in fractions regarding each individual MIC.

**Table 3:** Antibacterial activity of 35409-derived peptides against ATCC strains and *E. coli* clinical isolates. Peptide MIC and MBC are expressed in µM.

Peptide	MIC/MBC (ATCC Strains)			MIC/MBC (Clinical Isolates)		
	*E. coli ML35* 43827	*E. coli* 35218	*E. coli* 11775	*E. coli* N°4	*E. coli* N°40	*E. coli* N°44
**35409**	25/25	6/6	>100	-	-	-
**35409-1**	3/3	6/6	>100	>100	25	12.5/100
**35409-2**	6/12.5	12.5/25	>100	>100	50	50/>100
**35409-4**	25/50	25/50	>100	>100	>100	100/>100
**35409-13**	25/50	25/100	100/>100	>100	50/100	25/50
Ciprofloxacin *	0.008	0.016	0.016	>0.256	0.016	>0.256
Gentamicin *	-	-	-	2	1	>16
Ampicillin *	<0.016	>16	<0.016	-	-	-

* MIC in µg/mL.

**Table 4:** Antibacterial activity of 35409-derived peptides in the presence of fresh human sera.

Peptide	Sequence	MIC (µM) against *E. coli* 25922		
		MHB	Serum (without Pre-Incubation)	Serum (6 h Pre-Incubation)
**35409**	RYRRKKKMKKALQYIKLLKE	25	25	>100
**35409-1**	RKKKMKKALQYIKLLKE	25	12.5	50
**35409-2**	KKKMKKALQYIKLLKE	25	25	>100
**35409-4**	KMKKALQYIKLLKE	100	100	>100
**35409-13**	KKMKKALQYIKLLK	50	50	>100
Ciprofloxacin *		0.008	0.008	0.004

* MIC in µg/mL.

## Supplementary Materials

The following are available online at https://www.mdpi.com/2076-2607/8/6/867/s1. Figure S1: Calibration curves used with the bacterial strains in this study and their percentage variation. Some curves were used for more than one bacterial strain, as can be seen on this figure’s inset. The OD was measured in 270 µL volume. Figure S2: Data regarding the synthesis, purification and characterisation of the most representative peptide from this study (peptide 35409-1). A. Summary of synthesis from the C-terminal extreme to the N-terminal extreme. B. Pure peptide chromatographic profile (HPLC). C. MALDI-TOF mass spectra, the observed signal at 2144.6 corresponds to the ${\left[M+H\right]}^{+}$ specie. Table S1: RP-HPLC chromatographic characterisation of 35409 derived-peptides.

## Appendix A

***E. coli* Clinical Isolates**			**Isolate ID**	**4**	**40**	**44**
			**Sample Origin**	**Urine**	**Blood**	**Blood**
**Type of antibiotic**			**Antibiotic**	**MIC (µg/mL)**	**MIC (µg/mL)**	**MIC (µg/mL)**
**Resistance profile**	**Aminoglycosides**		**Amikacin**	≤2 (S)	≤2 (S)	32 (I)
			**Gentamicin**	≤1 (S)	≤1 (S)	≥16 (R)
	**β-lactamases**		**Ampicillin**	≥32 (R)	≥32 (R)	≥32 (R)
	**β** **-lactam +** **β** **-lactamase inhibitor**		**Ampicillin/sulbactam**	8 (S)	16 (I)	≥32 (R)
			**Piperacillin/Tazobactam**	≤4 (S)	≤4 (S)	≥128 (R)
	**β** **-lactamases (cephalosporins) +** **β** **-lactamase resistant**	**1st-generation**	**Cephalothin**	4 (S)	16 (I)	≥64 (R)
		**2nd-generation**	**Cefoxitin**	≤4 (S)	≤4 (S)	≤4 (S)
		**3rd-generation**	**Cefotaxime**	≤1 (S)	≤1 (S)	≥64 (R)
			**Ceftazidime**	≤1 (S)	≤1 (S)	≥64 (R)
		**4th-generation**	**Cefepime**	≤1 (S)	≤1 (S)	≥64 (R)
	**β-lactamases**	**Carbapenems**	**Imipenem**	≤1 (S)	≤1 (S)	≤1 (S)
			**Meropenem**	≤0.25 (S)	≤0.25 (S)	≤0.25 (S)
	**ESBL**			-	-	+
	**Quinolones**		**Ciprofloxacin**	≥4 (R)	≤0.25 (S)	≥4 (R)
			**Nalidixic acid**	≥32 (R)	≤2 (S)	≥32 (R)
	**Nitrofuran**		**Nitrofurantoin**	≤16 (S)	≤16 (S)	≤16 (S)
	**Co-trimoxazole**		**Trimethoprim/sulfamethoxazole**	≥320 (R)	≤20 (S)	≤20 (S)
