# Supplementary material for: Shorter Antibacterial Peptide Having High Selectivity for E. coli Membranes and Low Potential for Inducing Resistance
Source: Microorganisms. 2020 Jun 8;8(6):867. doi: 10.3390/microorganisms8060867 (PMC7356157; doi:10.3390/microorganisms8060867)
Supplement: Supplementary file 1 [file microorganisms-08-00867-s001.pdf]

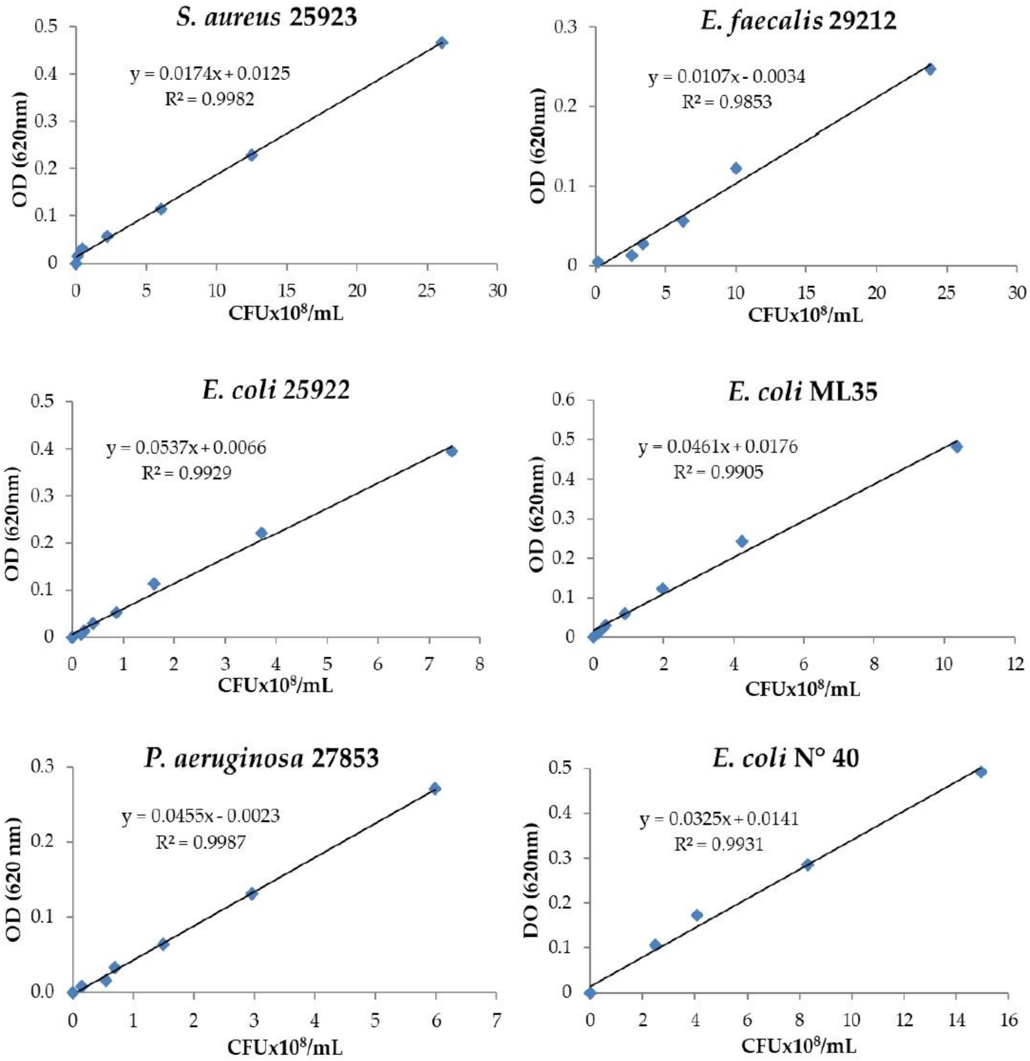

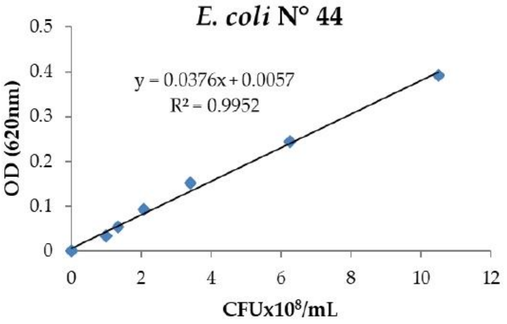

Strain/isolate	Curve equation	R^2	Variation (%)
S. aureus 25923	y = 0,0174x + 0,0125	0,9982	17
E. faecalis 29212	y = 0,0107x – 0,0034	0,9853	3
E. coli 25922	y = 0,0537x + 0,0066	0,9929	17
E. coli 35218	y = 0,0537x + 0,0066	0,9929	5
E. coli 11775	y = 0,0537x + 0,0066	0,9929	17
E. coli ML35 43827	y = 0,0461x + 0,0176	0,9905	3
P. aeruginosa 27853	y = 0,0455x – 0,0023	0,9987	2
E. coli N4	y = 0.0376x + 0.0057	0.9952	8
E. coli N40	y = 0.0325x + 0.0141	0.9931	14
E. coli N44	y = 0.0376x + 0.0057	0.9952	16

x= (CFU/mL)*10⁸; y=Absorbance

Figure S1: Calibration curves used with the bacterial strains in this study and their percentage variation. Some curves were used for more than one bacterial strain, as can be seen on this figure's inset. The OD was measured in 270 μL volume.

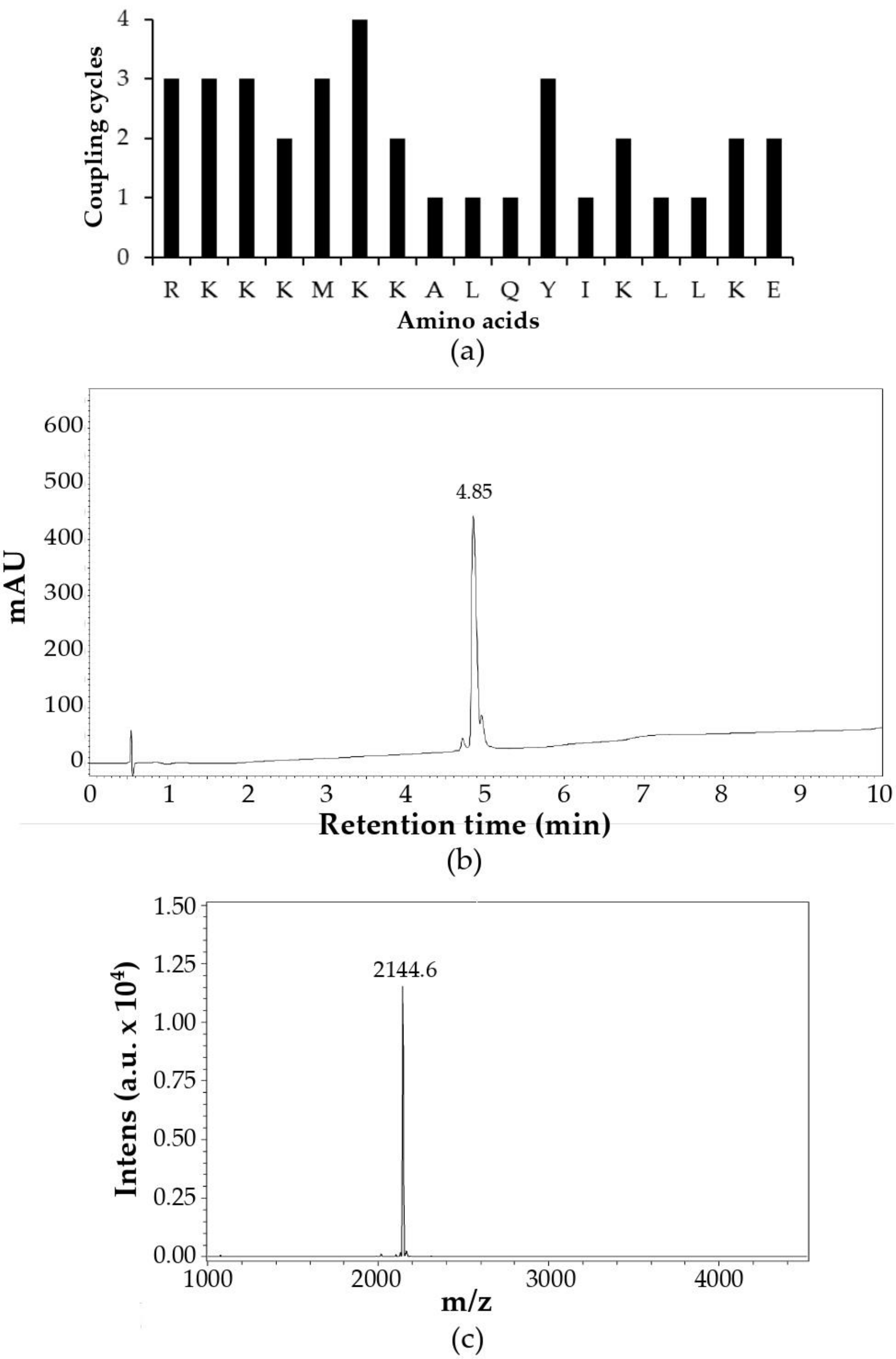

Figure S2: Data regarding the synthesis, purification and characterisation of the most representative peptide from this study (peptide 35409-1). A. Summary of synthesis from the C-terminal extreme to the N-terminal extreme. B. Pure peptide chromatographic profile (HPLC). C. MALDI-TOF mass spectra, the observed signal at 2144.6 corresponds to the $[M + H]^+$ specie.

Table S1. RP-HPLC chromatographic characterisation of 35409 derived-peptides

Peptide	Sequence	Length	Purity [a] (%) RP-HPLC
**35409**	RYRRKKKMKKALQYIKLLKE	20	96
**35409-1**	RKKKMKKALQYIKLLKE	17	86-95
**35409-2**	KKKMKKALQYIKLLKE	16	74
**35409-3**	KKMKKALQYIKLLKE	15	90
**35409-4**	KMKKALQYIKLLKE	14	71
**35409-5**	MKKALQYIKLLKE	13	98
**35409-6**	ALQYIKLLKE	10	99
**35409-7**	YIKLLKE	7	98
**35409-8**	RYRRKKKMKKALQYIKL	17	100
**35409-9**	RYRRKKKMKKALQY	14	98
**35409-10**	RYRRKKKMKKA	11	97
**35409-11**	RYRRKKKMKK	10	97
**35409-12**	RYRRKKK	7	90
**35409-13**	KKMKKALQYIKLLK	14	99
**35409-14**	KKMKKALQYIKL	12	97
**35409-15**	RKKKMKKALQY	11	98
**35409-16**	KMKKALQY	8	98

[a] Purity according to the percentage of the area below the chromatographic curve regarding the whole chromatogram. Two batches involving different synthesis, purification and characterisation processes were used for 35409-1 peptide, one having 86% chromatographic purity and the other 95%. No change in MIC results was observed between batches of the peptide.
